# Fish Sound Production in the Presence of Harmful Algal Blooms in the Eastern Gulf of Mexico

**DOI:** 10.1371/journal.pone.0114893

**Published:** 2014-12-31

**Authors:** Carrie C. Wall, Chad Lembke, Chuanmin Hu, David A. Mann

**Affiliations:** 1 College of Marine Science, University of South Florida, St. Petersburg, Florida, United States of America; 2 Center for Ocean Technology, University of South Florida, St. Petersburg, Florida, United States of America; 3 Loggerhead Instruments, Inc., Sarasota, Florida, United States of America; University of Windsor, Canada

## Abstract

This paper presents the first known research to examine sound production by fishes during harmful algal blooms (HABs). Most fish sound production is species-specific and repetitive, enabling passive acoustic monitoring to identify the distribution and behavior of soniferous species. Autonomous gliders that collect passive acoustic data and environmental data concurrently can be used to establish the oceanographic conditions surrounding sound-producing organisms. Three passive acoustic glider missions were conducted off west-central Florida in October 2011, and September and October 2012. The deployment period for two missions was dictated by the presence of red tide events with the glider path specifically set to encounter toxic *Karenia brevis* blooms (a.k.a red tides). Oceanographic conditions measured by the glider were significantly correlated to the variation in sounds from six known or suspected species of fish across the three missions with depth consistently being the most significant factor. At the time and space scales of this study, there was no detectable effect of red tide on sound production. Sounds were still recorded within red tide-affected waters from species with overlapping depth ranges. These results suggest that the fishes studied here did not alter their sound production nor migrate out of red tide-affected areas. Although these results are preliminary because of the limited measurements, the data and methods presented here provide a proof of principle and could serve as protocol for future studies on the effects of algal blooms on the behavior of soniferous fishes. To fully capture the effects of episodic events, we suggest that stationary or vertically profiling acoustic recorders and environmental sampling be used as a complement to glider measurements.

## Introduction

Passive acoustic monitoring (PAM) allows sound-producing animals to be non-invasively sampled for long periods of time. Soniferous fish use sound for communication that is typically associated with courtship, spawning, parental, territorial or aggressive behavior [1]. Most fish calls are low frequency (<3 kHz), species-specific and repetitive. The three most common sound producing mechanisms employed by fish are swimbladder muscle contractions (short duration (∼<5 s), low frequency (<1500 Hz) tones with multiple harmonics), stridulation (shorter duration (<1 s), broadband pulses up to a few kHz) [2]–[4], and air passage sounds (up to 10 kHz) [5]–[7]. These characteristics are markedly different from sounds produced by marine mammals, which are infrequent, range up to 200 kHz, and can be long in duration (>10 s) [8]. Passive acoustic monitoring of fishes has enabled researchers to identify fish species distribution and behavior using moored devices and, more recently, hydrophone-integrated autonomous gliders [9]–[15]. Gliders fitted with optical and environmental sensors [16]–[22] that concurrently collect data can be used to establish the oceanographic conditions surrounding sound-producing inhabitants [23]–[25].

Research using passive acoustic data collected by gliders examined large-scale and long-term fish sound production in the eastern Gulf of Mexico from red grouper, *Epinephelus morio*, toadfish, *Opsanus* sp. and previously undocumented sounds [24], [25]. Wall *et al.* [24] reported four new biological sounds likely produced by fishes and suggest potential sources. These sounds consist of: (1) “100 Hz Pulsing”, a series of clicks with an average peak frequency of 100 Hz suspected to be produced by cusk eel species (*Lepophidium* sp. and/or *Ophidion* sp.); (2) “6 kHz Sound”, a 200–500 Hz wide tone suspected to be related to gas release in clupeid schools, namely Atlantic thread herring (*Opisthonema oglinum*); (3) “300 Hz FM Harmonic”, a frequency modulated tonal harmonic with an average peak frequency of 300 Hz suspected to be produced from Atlantic midshipman (*Porichthys plectrodon*), and (4) “365 Hz Harmonic”, a tonal harmonic with a peak frequency of 365 Hz suspected to be from searobins (*Prionotus* sp.) [24]. Nomenclature used here derives from a characteristic feature. Harmonic refers to tonal sounds usually resulting from swimbladder muscle contraction that contain additional frequencies at an integer multiple of the fundamental frequency. A pulse is a very short duration, broadband sound and pulsing refers to pulses that repeat rapidly for a relatively long period of time (>1 s). Conversely, knocks applies to pulses that repeat for a shorter period of time (<1 s). The correspondence of these call characteristics to typical fish sounds (short duration, low frequency, and repetitive) supports the hypothesis that they are produced by fishes.

In the Gulf of Mexico, toxic blooms of the dinoflagellate, *Karenia brevis* (also called red tides), have occurred nearly annually since the 1840s [26] and are most common on the West Florida Shelf (WFS) in the late summer and fall [27]. Extended red tide events detrimentally impact numerous trophic levels, including significant fish kills, toxic shellfish poisoning, and respiratory problems in humans [28], [29]. Vargo [30] reviews environmental parameters responsible for the growth, distribution, and nutrient requirements of *K. brevis* and hypotheses on the mechanisms responsible for bloom initiation, maintenance, and termination. Despite the long history of red tide events and environmental impacts, details of their origination are disputed, and the non-lethal impacts on fishes and fish behavior remain largely unknown [31], [32], [29], [33], [30]. However, recent research that employs glider technology with integrated optical sensors to identify the 3-D structure and movement of *K. brevis* is helping to advance detection and monitoring of these blooms [34]–[36]. It is therefore desirable to use gliders to study whether and how red tides may impact fish behavior.

Here, we used passive acoustic and optical data recorded throughout three glider missions in the eastern Gulf of Mexico to determine the impact, if any, of *K. brevis* blooms on sound production from known (e.g., red grouper *Epinephelus morio* and toadfish *Opsanus* sp.) and suspected fish species. To our knowledge, this is the first work to examine fish sound production in the presence of red tides. As the original goal of these glider missions was to examine bio-optical conditions [36], this examination of concurrently collected passive acoustic data is the result of an opportunistic dataset. Our aim was to address a different scientific question using the data available to determine whether an effect of the red tide on sound-producing fishes was detectable.

## Materials and Methods

A hydrophone was integrated into the aft cowling of a Slocum electric underwater glider (Teledyne Webb Research) to record sound while simultaneously collecting a suite of environmental and optical parameters, including, water temperature (Temp), salinity (Sal), dissolved O_2_ (DO), chlorophyll *a* (Chl), color dissolved organic matter (CDOM), backscatter (β_(650 nm, 117°)_), depth in the water column, and bottom depth. Water column depth was determined using the pressure recorded by the glider's CTD (Seabird SBE 19Plus CTD), which also provided the temperature and conductivity information. An Aanderaa oxygen optode 3835 was used to measure the DO concentration. A WETLabs ECO Triplet measured optical scattering and fluorescence, with reported values derived using the manufacturer's calibration coefficients.

The glider ascends and descends the water column in a sawtooth-like profile as it moves along its pre-determined track. Latitude and longitude were collected via satellite link when the glider was at the surface. The position of the glider when not at the surface was estimated from the surface latitude and longitude coordinates using linear interpolation and a 10-point moving average.

Acoustic data were recorded using a Digital Spectrogram Recorder (DSG; Loggerhead Instruments, Inc.). The DSG is a low-power acoustic recording system controlled by script files stored on a 32 GB secure digital (SD) memory card and an on-board real-time clock (RTC). The RTC maintains accuracy with temperature-compensated drift. The DSG data structure associates RTC data with acoustic data that can be time synchronized to other glider data. Hydrophone (HTI-96-MIN, sensitivity −170 dBV, ±3 dB from 2 Hz to 37 kHz, High Tech, Inc.) signals were digitized with 16-bit resolution by the DSG recorder.

**Figure 1:**
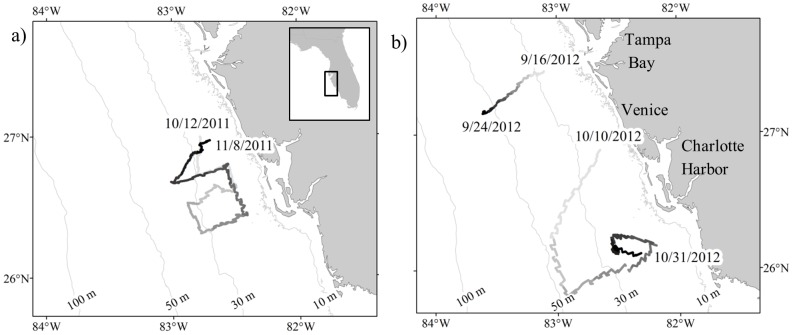
Location of glider deployments for a) Mission 54 in 2011, and b) Mission 56 (Sep 16–24) and Mission 57 (Oct 10–31) in 2012. Glider tracks are shown in grayscale with increasing time represented by increasing darkness. Deployment start and end dates are noted by their corresponding geographic location. The black box within the inset in a) illustrates the study area located off west-central Florida.

Three glider missions (Mission 54, 56, and 57) were conducted in the eastern Gulf of Mexico in October 2011, and September and October 2012 (Fig. 1). Gliders were run and maintained by the Center for Ocean Technology at the University of South Florida, College of Marine Science. No specific permissions were required for these locations or activities as all samples were collected passively outside of protected waters and without direct, intended approach, interference, or inclusion of endangered or protected species. The deployment location and period for Missions 54 and 57 were dictated by the presence of red tide-affected waters off west-central Florida. Mission 56 occurred when red tide was not expected to be present. Mission 56, supplemented by the previously documented work of Wall *et al.* [24], serves as a baseline for fish sound production. The DSG recorded sound for 15 s every 5 min at a sample rate of 70 kHz during Missions 54 and 56. Due to the longer deployment period, the DSG recorded sound for 10 s every 5 min during Mission 57.

All acoustic files were analyzed audibly and visually to identify the presence of fish sounds. Visual analysis was performed on spectrograms created in MATLAB (Mathworks) using 2,048 point Hanning-windowed FFTs with 50% overlap. Due to the frequent occurrence of overlapping calls, both intrapecific and interspecific, each fish sound was documented as either present or absent within an acoustic file, representing a 10 or 15 s time period. The percentage of time recorded was lower for Mission 57 due to the 10 s recording duration, compared to 15 s for the other missions. This disparity is unlikely to significantly affect the results due to the fact that fish call repeatedly for hours on end, especially at night. If a fish calls repeatedly for 6 hours, the difference between recording for 10 s or 15 s becomes negligible as the sound will still be captured in either scenario. This is further supported by an examination of 100 randomly chosen 15 s files recorded at night during Mission 56. No files contained fish sound(s) present only in the last 5 s.

The acoustic files were analyzed specifically for the presence of eight known or suspected fish sounds: red grouper, toadfish, 6 kHz Sound, 300 Hz FM Harmonic, 365 Hz Harmonic, 100 Hz pulsing, “Cusk eel”, and “360 Hz Knocks”. The latter two sounds were not reported in Wall *et al.* [24] but were commonly observed throughout this study. The Cusk eel sound has been verified to be similar to the documented sound production of striped cusk eel *Ophidion marginatum* [37], [38]. Although striped cusk eel are not present in the eastern Gulf of Mexico, it is with high certainty that this sound is produced by a similar species. This sound and 360 Hz Knocks are described in detail in the Results section.

The call characteristics and distribution on the WFS are already known for all sounds except Cusk eel and 360 Hz Knocks. Wall *et al*. [24] determined that across a study area with bottom depths ranging from 4 to 984 m and over a one year period, the 6 kHz Sound and the 365 Hz Harmonic sounds are most common in depths shallower than 40 m, whereas the 300 Hz FM Harmonic sounds are found in offshore waters (50–200 m bottom depths). Toadfish and the 100 Hz Pulsing are observed throughout a variety of bottom depths (<10 m to 100 m) [24]. Outside of hard bottom and offshore marine protected areas, red grouper sounds are most common between 20 and 50 m, [25]. Wall *et al.* [24], [25] identified that all but red grouper and toadfish chorus (very frequent calling by multiple individuals) at night. Conversely, red grouper call throughout a 24 hr period with a slight decrease in calling between sunset and sunrise [14], [25]. Throughout a 24 hr period, toadfish calling only decreases around sunrise [24].

### Oceanographic Data

Environmental and optical conditions, glider depth in the water column, and bottom depth were extracted at the time of each acoustic file. Since the known fish sounds (red grouper and toadfish) and suspected sources of the 100 Hz pulsing (cusk eel sp.), Cusk eel (cusk eel sp.), 300 Hz FM Harmonic (Atlantic midshipman), and 365 Hz Harmonic (searobin sp.) are all demersal species, oceanographic data recorded near the seafloor were used for the analysis as they are most indicative of the conditions at the sound source. Although the exact depth in the water column of the suspected source of the 6 kHz Sound – pelagic Clupeids – is unknown, for consistency and in order to simplify the statistical analysis, data collected at depth were also used for this sound. Since the glider was programmed to descend and ascend without contacting the seafloor, near-seafloor conditions for each fish sound was determined using the nearest chronological environmental data point that was recorded at approximately 3 m above the seafloor (median ±SD: 2.8 m above the seafloor ±5.1, N = 15,409).

The red tide event in 2011 that was captured in Mission 54 has been characterized by Zhao *et al.* [36]. By incorporating fluorescence line height (FLH) and Red-Green-Blue (true color) satellite images collected by the Moderate Resolution Imaging Spectroradiometer (MODIS) and Medium Resolution Imaging Spectrometer (MERIS), and *in situ* glider-derived optical and *K. brevis* data, Zhao *et al.* [36] established a synoptic, 3-D understanding of the bloom. The main optical characteristics associated with the 2011 red tide-affected waters were high Chl and CDOM. Since the red tide captured in Mission 57 has not been previously documented, we used the same methods as Zhao *et al.* [36] to determine when the glider was likely in the bloom. Here, blooms were characterized as having increased (>5) Chl and CDOM glider optical data values near the seafloor and high (>0.03∼0.05) FLH values.

### Statistical Analysis

Fish sound detections were binned by hour (presence out of 12 files, equivalent to 60 min), and the median value of the optical data, environmental data, bottom depth and latitudinal position of the glider each hour were calculated. It should be noted that the bottom depth variable was expressed as a negative altitude (i.e., smaller values indicate deeper water). A red tide variable was calculated as a binary index where 1 corresponds to when the glider was in red-tide affected waters (based on elevated,>5, Chl and CDOM values, and confirmed by FLH satellite imagery) and 0 corresponds to no red tide. This variable also allowed for easy separation of sounds that were recorded inside the bloom and those that occurred outside for further comparisons.

As six out of the eight fish sounds presented here are nocturnal, only sounds recorded at night (19:00 h to 7:00 h local) were incorporated into the statistical analyses. This restriction removed the well-established nocturnal chorusing from dominating the results. Since red grouper and toadfish call over a 24 hr period they will still be represented in the night data.

A redundancy analysis (RDA) was applied to the z-scores of each mission's acoustic and oceanographic data to determine how much variability in the response variables (presence of each sound binned hourly) can be explained by the predictor variables (associated oceanographic data binned hourly). RDA linearly models the multivariate response variables against the multivariate predictor variables, and then applies a principal component analysis to the results to extract the eigenvectors and eigenvalues [39]–[41]. The eigenvectors, constrained as a linear combination of the predictor variables, are used to calculate scores for each object and derive scaling/ordination plots [40]. These results are displayed as biplots where the longest predictor vectors are most important in explaining variation in sound production. The proximity between the predictor vectors and the response variables reflect their correlation (closer proximity indicates stronger correlation). A Hellinger transform was applied to the response variables, which allows RDA to be used on species data [42], [43]. 1,000 iterations of the permutation test were applied to the RDA. Analyses were completed in MATLAB (Mathworks) using the Fathom Toolbox [44].

## Results

A total of 7,479, 2,156, and 5,774 files were recorded in Mission 54, 56, and 57, respectively. Details on each mission, including duration, distance traveled, and bottom depth are outlined in Table 1 and the transects, which indicate the specific locations of study, can be seen in Fig. 1. Mission 54 was deployed off Venice, FL in 17 m bottom depth. An S-shaped glider track resulted in a variety of depths and latitudes surveyed as the glider moved offshore and onshore, and along isobaths. The glider was recovered northwest of Charlotte Harbor on November 8, 2011. Mission 56 had the shortest duration, simplest track, and deepest depth range of all the missions. The glider was deployed 4 km southwest of the mouth of Tampa Bay in 27 m bottom depth where it continued southwest to 55 m bottom depth when it was recovered on September 24, 2012. Mission 57 was deployed off Venice, FL in 16 m bottom depth. The glider then headed southwest to 50 m bottom depth before turning northeast. The glider made negative progress from October 26 to 29 due to storm events and high currents causing the track to move south instead of the intended north/northwest transit. On October 31, 2012 the glider was recovered southwest of Charlotte Harbor.

**Table 1:** Glider deployment information for Missions 54, 56, and 57.

Mission	Deployed	Recovered	Days	Dist. (km)	Min. Depth (m)	Max. Depth (m)	Files Recorded	In Red Tide (%)
54	10/12/2011	11/8/2011	28	385	17	39.1	7,479	15.04
56	9/16/2012	9/24/2012	8	70	27	55	2,156	NA
57	10/10/2012	10/31/2012	21	350	16	50	5,774	14.77

The percentage of files recorded when the glider was in red tide are provided. NA denotes not applicable.

**Figure 2:**
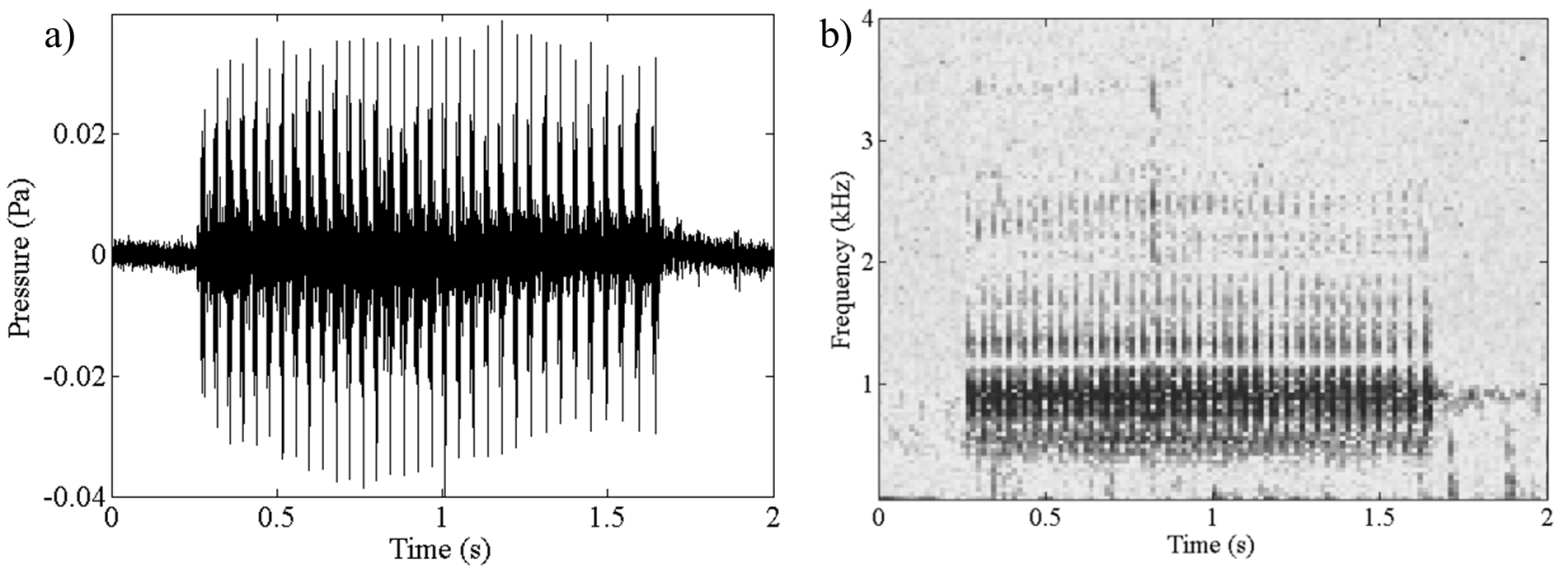
Cusk eel sound production illustrated as a) waveform and b) spectrogram. An example of this recording is provided in the Supporting Information (S1 Fig.).

The two previously undocumented sounds are described as follows. “Cusk eel” sounds consist of a series of pulses with a mean fundamental frequency of 650 Hz (110 Hz SD, n = 15), a peak frequency of 900 Hz (60 Hz SD, n = 15), and harmonics reaching 6,500 Hz (Fig. 2). Fast repetitive pulses, or pulse trains, lasted from 1 to 4 s. Chorusing was observed during the day and night with the majority of sounds occurring after sunset (79.7% of the files containing this sound were recorded between 19:00 h and 7:00 h; 1,514/1,880 files). This sound was recorded in water up to 35 m bottom depth (88% of the files containing this sound were associated with less than 25 m bottom depth; 1,654/1,880 files). An example of this sound is provided in the Supporting Information (S1 Fig.).

**Figure 3:**
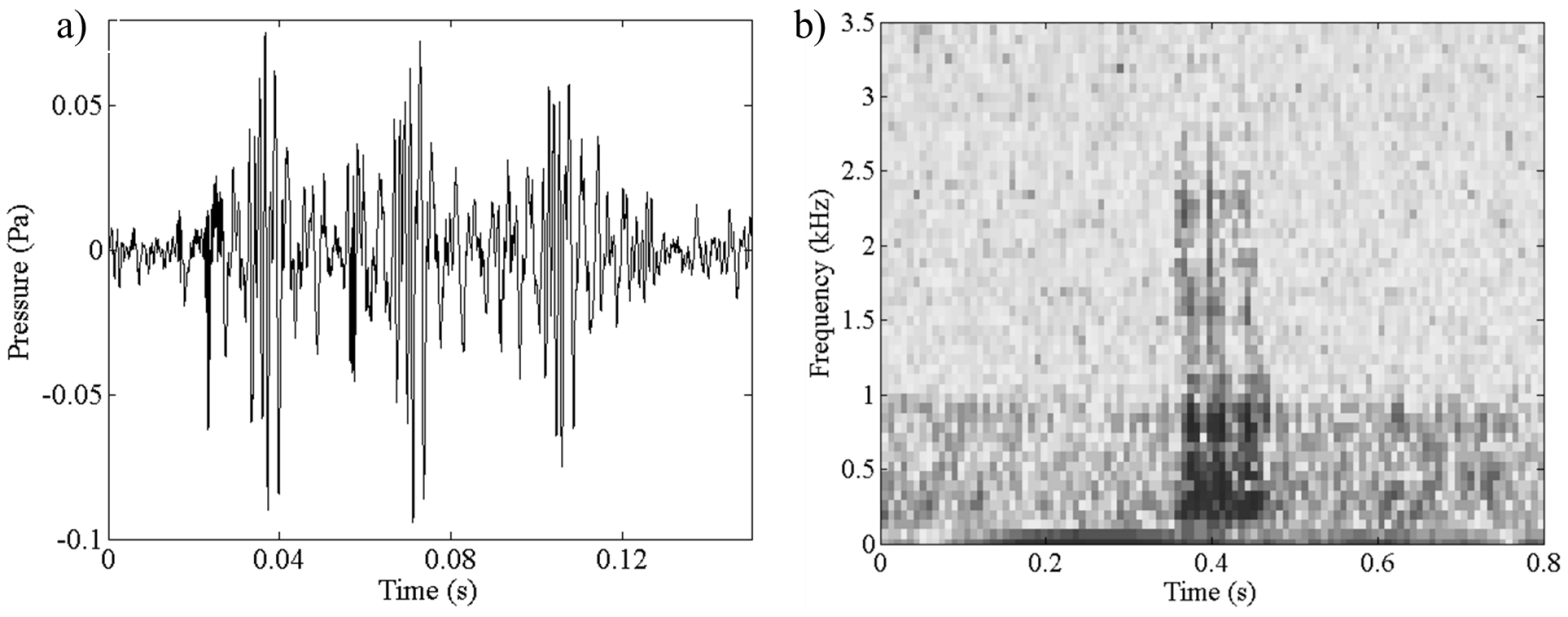
360 Hz Knocks sound example illustrated as a) waveform and b) spectrogram. An example of this recording is provided in the Supporting Information (S2 Fig.).

The 360 Hz Knocks also consist of a series of pulses and have a 360 Hz peak frequency (Fig. 3). The fundamental frequency of this sound is 100 Hz with energy reaching upwards of 4,400 Hz. Mean call duration (120±10 ms, n = 28), pulse duration (20±2 ms, n = 28), and inter-pulse interval (10±2 ms, n = 21) were also calculated. Pulse trains typically consisted of three pulses, however, two to four pulses were observed in some recordings. This sound was nocturnal (91.3% of the files containing this sound were recorded between 19:00 h and 7:00 h; 928/1,016 files) and occurred in shallow water (92% of the files containing this sound were associated with less than 35 m bottom depth; 931/1,016 files) with no observations beyond 48 m bottom depth. An example of this sound is provided in the Supporting Information (S2 Fig.). The correspondence of the call characteristics for these two sounds to typical fish sounds leads us to hypothesize that they are produced by fishes.

The percentage of files containing sounds produced by red grouper, toadfish, 100 Hz pulsing, 6 kHz Sound, 300 Hz FM Harmonic, 365 Hz Harmonic, Cusk eel, and 360 Hz Knocks are outlined in Table 2. The 6 kHz Sound was the most common sound identified throughout the study, occurring in 28%, 26%, and 22% of the files recorded in Mission 54, 56, and 57, respectively. Cusk eel sounds were the next most common with 17% and 11% of the files identified in Missions 54 and 57, respectively, but was rare (<1%) in Mission 56. Red grouper sounds were most commonly identified in Mission 56 (23%) with decreasing occurrence in Mission 57 (13%) and Mission 54 (3%). Similarly, the 300 Hz FM Harmonic was commonly identified in Mission 56 (30%) with only rare occurrences in the other two missions (<3%). The 100 Hz pulsing and 360 Hz Knocks showed similar occurrence across all missions (6–9%). Toadfish sounds were consistently rare (<1%) throughout the study. Due to the low occurrence of toadfish and the 300 Hz FM Harmonic, specifically within Missions 54 and 57, these sounds were not included in further analyses.

**Table 2:** The percentage of files containing categorized sounds.

Mission	RG	Toadfish	100 Hz Pulsing	6 kHz Sound	300 Hz FM Harm.	365 Hz Harm.	Cusk eel	360 Hz Knocks
54	3.3	0.03	5.4	28	0.01	14	16.9	6.2
	(1.6)	(0)	(5.7)	(20.0)	(0)	(16.1)	(44)	(15.6)
56	22.8	0.3	5.9	25.5	29.6	12.5	0.1	1.5
	(NA)	(NA)	(NA)	(NA)	(NA)	(NA)	(NA)	(NA)
57	13.2	0.1	7.7	21.7	2.7	9.4	10.7	8.9
	(0.1)	(0)	(5.5)	(32.5)	(3.1)	(0)	(49.7)	(12.2)
**N**	**1,505**	**11**	**994**	**3,895**	**793**	**1,856**	**1,880**	**1,016**

The numbers in parenthesis indicate the percentage of files containing the associated sound recorded when the glider was in red tide-affected waters. NA denotes not applicable. The number of files (N) containing each sound combined for all missions is shown in bold.

### Oceanographic Data

**Figure 4:**
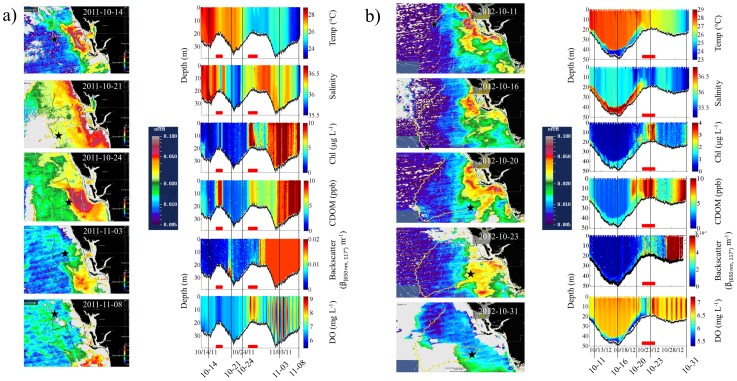
Red tide events during a) Mission 54 in 2011 and b) Mission 57 in 2012. (left) Plots of the glider track (grey or yellow lines) and position (black star) overlaid onto FLH satellite images (color scale) and currents (white vectors). High FLH values off Charlotte Harbor delineate phytoplankton blooms, confirmed to be *K. brevis* red tides from water sample analysis [36]. (right) Physical, chemical, and bio-optical data collected by the glider with demarcations for dates of shown satellite images. Red bars indicate time period when the glider is suspected to be in red tide affected waters. Note the difference in scales.

Plots of the glider data and associated FLH satellite images with overlaid glider tracks illustrate the surface to depth environment as measured by the glider and the location of the glider in relation to synoptic surface characteristics (Fig. 4). Based on both the glider optical data and satellite imagery, the time periods for the two missions in which the glider was suspected to be in red tide-affected water were: October 16 to 17 and 25 to 28, 2011 for Mission 54 and October 21 to 24, 2012 for Mission 57. The zig-zag movement of the glider throughout Mission 54 caused it to reach the outer edges of the red tide bloom for a brief period of time before moving offshore again (Fig. 4a). Once the glider moved back inshore, it re-entered and traversed through the bloom for several days before moving far enough north that it exited red tide-affected waters.

Unlike the relatively homogenous waters encountered in Mission 54, a strong thermocline 5 to 10 m off the seafloor was observed in Mission 57 from October 10–22. Waters below this thermocline were cold and contained high salinity, low DO, low CDOM, and periodic chlorophyll blooms (high Chl) (Fig. 4b). In the shallow, well-mixed water south of Charlotte Harbor lies the bloom. A strong thermocline was observed in Mission 56, especially beyond 30 m depth, with colder waters reaching up approximately 10 m above the seafloor (S3 Fig.). Waters below the thermocline contained high salinity, low DO, and high CDOM with periodic chlorophyll blooms. The banding pattern of DO in the missions illustrates the diurnal effects of photosynthesis and respiration.

Biofouling on the fluorometer began significantly impacting backscatter and other optical measurements on approximately October 30, 2011 in Mission 54 and October 27, 2012 in Mission 57, roughly 18 days after the start of each mission. Statistical analyses for these missions were limited to the time period prior to the onset of severe biofouling. No biofouling was observed during Mission 56 due to the shorter deployment period.

Approximately 15% of the acoustic files were recorded when the glider was in red tide-affected waters (see Table 1). The percentage of files in which each sound was recorded when the glider was in red tide-affected waters is included in Table 2. Very few red grouper and 100 Hz Pulsing sounds (<6% of the files) were recorded in the blooms. In comparison, 50% of the Cusk eel sounds and 20–33% of the 6 kHz Sounds were recorded at this time. The 365 Hz Harmonic and 360 Hz Knocks had a marginal presence (∼16%) when the glider was in red tide water in Mission 54. During Mission 57, no (0%) 365 Hz Harmonic sounds and approximately 12% of the 360 Hz Knocks were recorded in the bloom.

### Statistical Results

**Figure 5:**
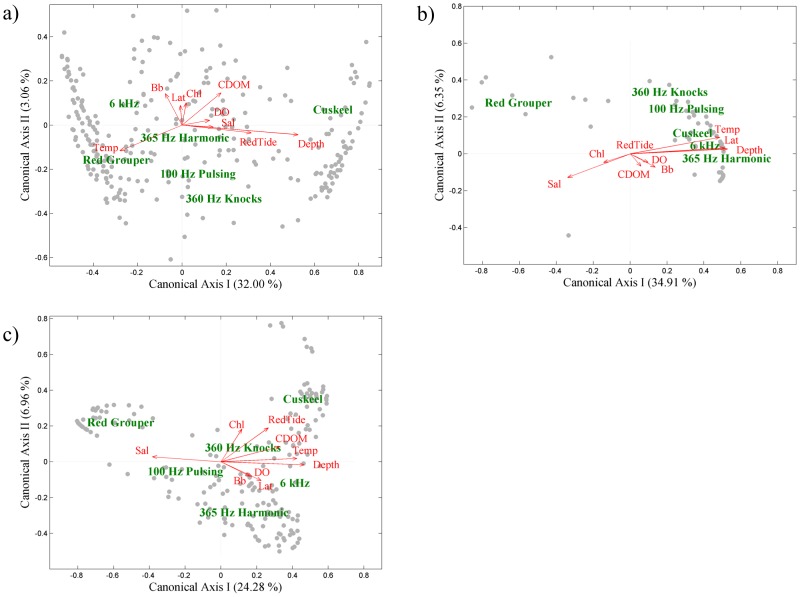
Results from the RDA for a) Mission 54, b) Mission 56, and c) Mission 57. The response variables (fish sound production) are labeled in green. The predictor variables (oceanographic data) are labeled in red next to their corresponding vector that indicates the direction and strength of their correlation to the response variables. Vector length is proportional to importance in explaining variation in response variables. Grey circles are each response variable displayed in RDA space. The proximity between the predictor vectors and the response variables reflect their correlation (closer proximity indicates stronger correlation). Due to close proximity and overlap with other vectors, some vectors may be difficult to discern. Bb represents the backscatter (β_(650 nm, 117°)_) variable.

Each mission identified a significant effect of oceanographic conditions on fish sound production (*p*<<0.01) (Table 3). Approximately 36%, 41%, and 34% of the variation in sounds detected was explained in Mission 54, 56, and 57, respectively. The strength of the relationship between the oceanographic parameters and sound production is described by the Species-Environment Correlation statistics (r), expressed by each canonical axis. These values indicate the influence an oceanographic parameter has in explaining variation in sound production. The biplots of the RDA results illustrate the correlation of the oceanographic variables (predictor) to sound production (response) (Fig. 5). Vector length is proportional to importance in explaining variation in response variables.

**Table 3:** RDA results for Missions 54, 56, and 57.

Mission	F	*p*	R^2^	r					
54	22.80	0.001	0.36	0.83	0.49	0.41	0.25	0.15	0.09
56	80.41	0.001	0.41	0.80	0.62	0.50	0.41	0.27	0.08
57	16.07	0.001	0.34	0.81	0.67	0.53	0.36	0.22	0.13

The F-statistic (F), alpha (*p*), variance explained (R^2^), and species-environment correlations (r) are provided.

In Mission 54, bottom depth, water temperature, and red tide index contain the strongest correlation to sound production (Fig. 5a). Data clustering indicates that Cusk eel were more strongly and positively influenced by the red tide and depth variables, while red grouper was positively influenced by temperature and negatively influenced by red tide and depth. The RDA results for Mission 56 indicate that bottom depth, latitude, and temperature explain the most variation in sound production (Fig. 5b). Due to the presumed absence of red tide, the red tide variable vector does not deviate from the origin. Sound production from the 360 Hz Knocks, 100 Hz Pulsing, Cusk eel, 6 kHz Sound, and 365 Hz Harmonic are clustered together, and positively influenced by depth, latitude and temperature. Red grouper sound production was negatively influenced by these variables indicated by its position opposite to these vectors. Lastly in Mission 57, salinity, bottom depth, and temperature, followed very closely by CDOM and red tide, were most strongly correlated to sound production (Fig. 5c). The red tide vector again positively influenced Cusk eel sound production while salinity positively influenced red grouper.

**Figure 6:**
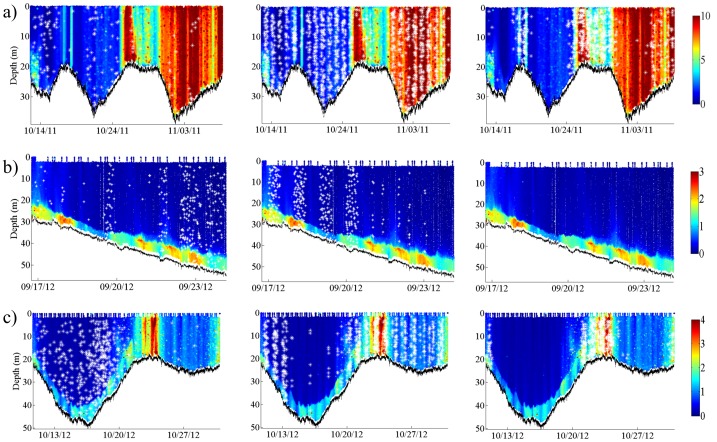
Profiles of Chl for a) Mission 54, b) Mission 56, and c) Mission 57 overlaid with (left) red grouper, (middle) 6 kHz Sound, and (right) Cusk eel sounds identified throughout each mission. For illustrative purposes, data points for each sound (white asterisks) are plotted based on the file timestamp and depth of the glider in the water column. Note the difference in scales; units are µg L^−1^.

**Figure 7:**
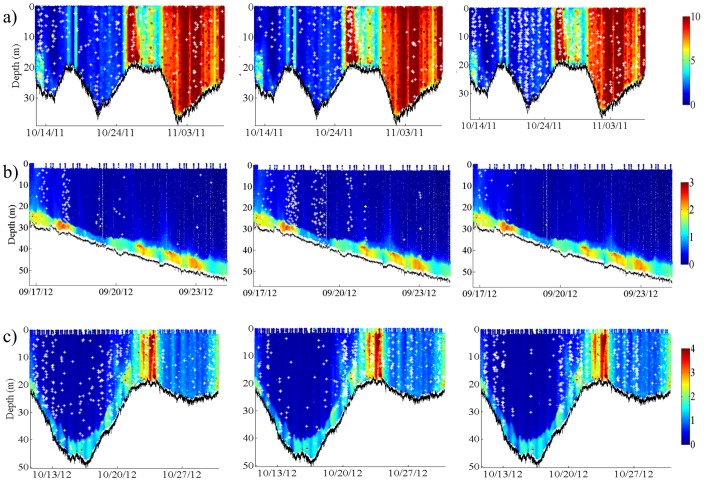
Profiles of Chl for a) Mission 54, b) Mission 56, and c) Mission 57 overlaid with (left) 100 Hz Pulsing, (middle) 365 Hz Harmonic, and (right) 360 Hz Knocks sounds identified throughout each mission. For illustrative purposes, data points for each sound (white asterisks) are plotted based on the file timestamp and depth of the glider in the water column. Note the difference in scales; units are µg L^−1^.

Red grouper, 6 kHz Sound, and Cusk eel sound production are plotted on top of the glider Chl data for each mission to illustrate their spatio-temporal occurrence with an indicator of red tide (Fig. 6). Red grouper calling was most common when the glider was in deeper water (>20 m depth) while Cusk eel sounds were most common in shallow waters (<20 m depth). The 6 kHz Sound was recorded when the glider was in water up to 40 m deep. The apparent banding of the 6 kHz Sound corresponds to the strong nocturnal occurrence of this sound [24]. The spatio-temporal patterns for 100 Hz Pulsing, 365 Hz Harmonic, and 360 Hz Knocks illustrate the nocturnal banding as well as the depth ranges of these sounds (Fig. 7). 100 Hz Pulsing was observed in an overall deeper depth range than 365 Hz Harmonic and 360 Hz Knocks, which were most common near shore (<35 m depth).

## Discussion

This paper examined sound production by fishes during red tide events in the eastern Gulf of Mexico. Oceanographic conditions measured by the glider were significantly correlated to the variation in fish sounds observed across the three missions. The environmental parameter that consistently contributed the greatest to the variation in fish sound production was bottom depth. The red tide index also contributed to sound production in Missions 54 and 57; however, this variable cannot be de-coupled from the bathymetry as the red tide-affected waters were confined to shallow depths (<25 m). Of particular note is the continued occurrence of sound production by most species within red tide-affected waters, namely nearly 50% of the Cusk eel sounds. Throughout both red tide events, DO remained above levels (<3 mg L^−1^) that commonly affect fishes [45], [46]. The lack of hypoxic conditions observed during the deployments may contribute to persistent fish sound production.

The range of bottom depths traversed across the three missions varied with Mission 56 containing the deepest depth range. The depth ranges of 100 Hz Pulsing, 6 kHz Sound, and 300 Hz Harmonic observed in this study were consistent with the expected depths documented in Wall *et al.* [24]. The low occurrence of red grouper sound production in red tide areas is attributed to this species' relatively deeper depth range [25]. These results suggest that the fishes encountered did not alter their call behavior nor migrate outside of expected depth ranges in the presence of red tide.

The latitude and temperature variables that were correlated to sound production arise from their correlation to bottom depth. The southwest direction of the glider throughout Mission 56 resulted in a consistently increasing bottom depth with decreasing latitude. In Mission 57, the cold water trapped below the thermocline resulted in decreased temperatures at deeper depths. The connection of sound production to the physical environment supports the influence and importance of abiotic factors on living marine resources and the overall marine ecosystem [47].

Determining when the glider was in red tide waters was done to the best of our ability; however, the complexity of these 3-D structures and the likelihood of occurrence at depth, out of sight from the surface satellite imagery, may result in conservative estimates. The examination of near seafloor optical conditions collected by the glider aimed to take this limitation into account. Without corresponding *in situ* sampling of *K. brevis* near the bottom, the timeline we estimated for when the glider was in the bloom and the expectation that no red tide was present during Mission 56 remain educated guesses. The current use of satellite imagery and near-shore sampling for monitoring may benefit from more regular vertical profiling across the shelf to better understand the composition of Chl blooms at depth throughout the year. The continued occurrence of fish sound production recorded in expected red tide blooms suggests that an extended bloom at depth, greater than accounted for here, would not affect the results of this work.

The 300 Hz FM Harmonic sound was observed only rarely in the recordings due to the most common occurrence of this sound in offshore waters (50–200 m bottom depths) [24] beyond the range of the three glider missions. While toadfish (*Opsanus* sp.) are observed throughout a variety of bottom depths (10–100 m), their boatwhistle calls are uncommon from August to March [24]. This reduction in sound production in the fall and winter explains the rare occurrence of toadfish sounds in the September and October glider missions presented here.

Two new sounds suspected to be produced by fish were identified in acoustic recordings: Cusk eel and 360 Hz Knocks. The call characteristics for Cusk eel support the potential source to be *Lepophidium* or *Ophidion* sp. [37], [38], [48], [49]. In the eastern Gulf of Mexico, Ophiidiformes are likely to produce sound nocturnally during the spawning season, which occurs in the fall [50], [24]. A more complicated puzzle is determining the source of the 360 Hz Knocks sound. There are nearly 90 genera of fishes in the Gulf of Mexico that are potentially soniferous [15]. Based on the repetitive, broadband pulsing, nocturnal chorusing, and relatively shallow occurrence (<35 m bottom depth), a member of the soniferous family Sciaenidae is a likely candidate, e.g., Atlantic croaker (*Micropogonius undulates*) or *Equetus* sp. [2], [51], [52]. Further fieldwork is needed to determine the source of these sounds with any certainty.

While the spectrum of vocalization repertoires is not known for all fish species including the sources of the unknown sounds, it is very unlikely for the same species to produce sounds in a different frequency range over a short period of time (i.e., produce calls between 100–130 Hz and also 350–400 Hz). Significantly different sounds in the same frequency range are possibly produced by the same species [53] but that is unlikely. Due to the large number of genera potentially producing sound, it is more likely for different fish species to make sound than for the same species to make two different sounds [1], [2]. Therefore, it is with high likelihood that the repetitive species-specific sounds analyzed here represent the true spectrum of the vocalization repertoire encountered. Further, these sounds embody the overwhelming majority of biological sounds recorded in the overall frequency range typical for fish (50 Hz–6 kHz).

The advantage of glider technology being able to cover a large spatial area is, in this case, also a drawback. The constantly moving gliders resulted in data that varied temporally and spatially. This limitation does not negate the importance of the relationships identified here, but complicates the interpretation and could be responsible for muted effects of red tide observed on fish sound production. At the time and space scales of these glider missions, there is no unequivocal, direct effect of red tide on sound production. This does not mean that effects will not be present one week later. Corresponding recordings from stationary observing stations would be a nice complement to the glider data to enable a direct comparison of behavior before and after the event, and capture delayed effects [54], [55], [49]. Hydrophone-integrated ocean profilers also have great potential to monitor long-term environmental conditions and identify episodic events present throughout the entire water column. Alternatively, if the glider or multiple gliders were deployed from near the shore to the shelf break and repeatedly sampled the same transect, a dataset with more controlled variables would result and a stronger comparison before and after the event would be possible. Since this research was conducted out of an opportunistic dataset, a deployment with multiple gliders and repeated transects or stationary recorders was not feasible. Further examination of a larger dataset using the methods presented here in concert with the additional observations proposed above is suggested protocol for future studies on the effects of harmful algal blooms on the behavior of soniferous fishes.

## Supporting Information

S1 Fig **Example sound file for Cusk eel.**
(WAV)

S2 Fig **Example sound file for 360 Hz Knocks.**
(WAV)

S3 Fig **Physical, chemical, and bio-optical data collected by the glider during Mission 56.**
(TIF)
