# Correction: Fish Sound Production in the Presence of Harmful Algal Blooms in the Eastern Gulf of Mexico

**DOI:** 10.1371/journal.pone.0121038

**Published:** 2015-03-20

**Authors:** The PLOS ONE Staff

The following information is missing from the Funding section: Publication of this article was funded by the University of Colorado Boulder Libraries Open Access Fund and Loggerhead Instruments.
