# Supplementary figures and images for: Fish Sound Production in the Presence of Harmful Algal Blooms in the Eastern Gulf of Mexico
Source: PLoS One. 2014 Dec 31;9(12):e114893. doi: 10.1371/journal.pone.0114893 (PMC4281131; doi:10.1371/journal.pone.0114893)

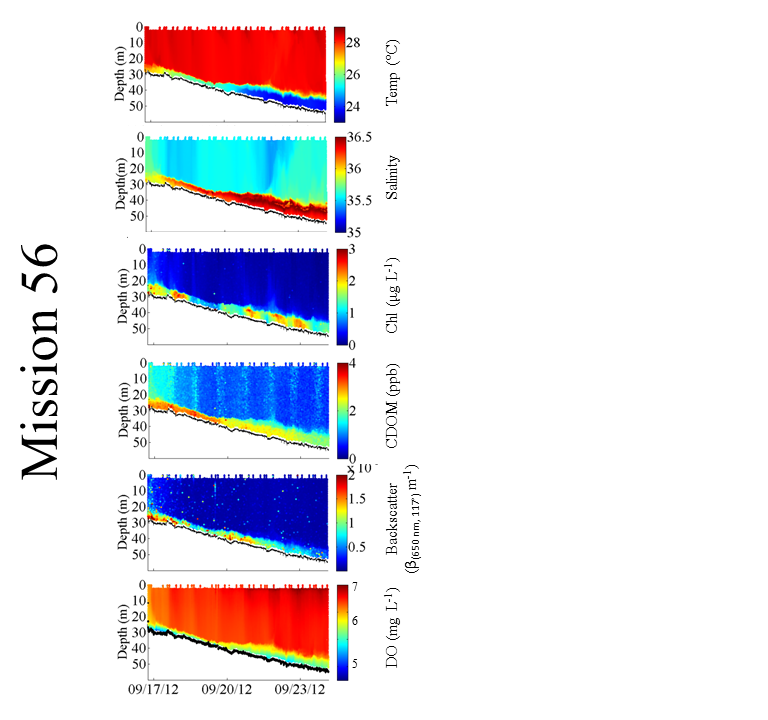

Supplement: S3 Fig — Physical, chemical, and bio-optical data collected by the glider during Mission 56. (TIF) [file pone.0114893.s003.tif]
